# Tracking ^14^C-Labeled Organic Micropollutants to Differentiate between Adsorption and Degradation in GAC and Biofilm Processes

**DOI:** 10.1021/acs.est.1c02728

**Published:** 2021-07-27

**Authors:** Alexander Betsholtz, Stina Karlsson, Ola Svahn, Åsa Davidsson, Michael Cimbritz, Per Falås

**Affiliations:** † Department of Chemical Engineering, Lund University, 221 00 Lund, Sweden; ‡ Sweden Water Research AB, Ideon Science Park, Scheelevägen 15, 223 70 Lund, Sweden; § School of Education and Environment, Division of Natural Sciences, Kristianstad University, 291 88 Kristianstad, Sweden

**Keywords:** pharmaceuticals, ^14^C-labeling, granular activated carbon, biofilms, transformation

## Abstract

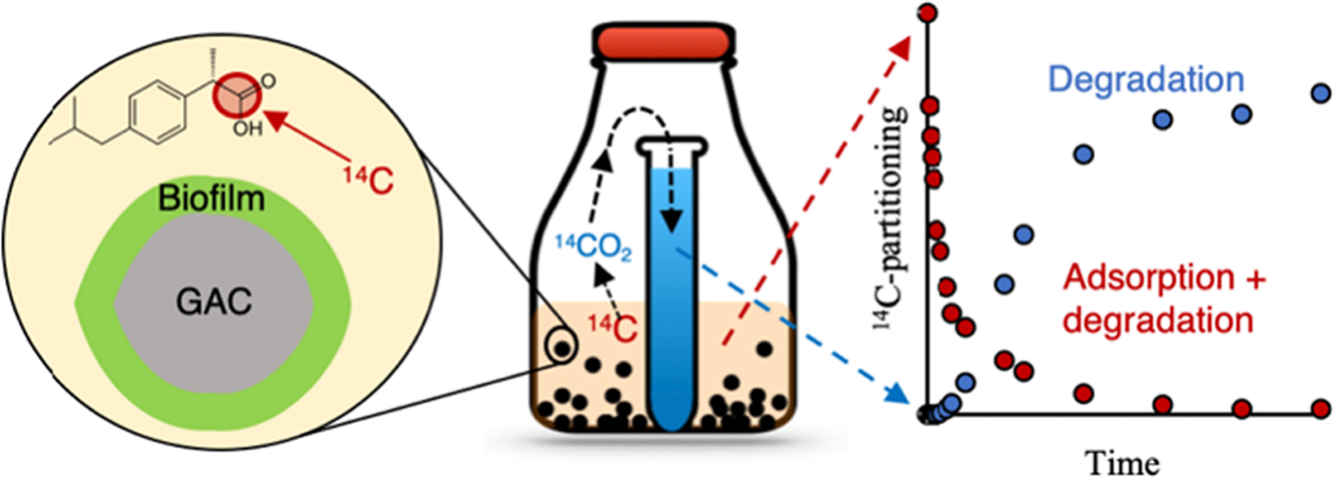

Granular activated carbon (GAC) filters can be used to reduce emissions of organic micropollutants via municipal wastewater, but it is still uncertain to which extent biological degradation contributes to their removal in GAC filters. ^14^C-labeled organic micropollutants were therefore used to distinguish degradation from adsorption in a GAC-filter media with associated biofilm. The rates and extents of biological degradation and adsorption were investigated and compared with other biofilm systems, including a moving bed biofilm reactor (MBBR) and a sand filter, by monitoring ^14^C activities in the liquid and gas phases. The microbial cleavage of ibuprofen, naproxen, diclofenac, and mecoprop was confirmed for all biofilms, based on the formation of ^14^CO_2_, whereas the degradation of ^14^C-labeled moieties of sulfamethoxazole and carbamazepine was undetected. Higher degradation rates for diclofenac were observed for the GAC-filter media than for the other biofilms. Degradation of previously adsorbed diclofenac onto GAC could be confirmed by the anaerobic adsorption and subsequent aerobic degradation by the GAC-bound biofilm. This study demonstrates the potential use of ^14^C-labeled micropollutants to study interactions and determine the relative contributions of adsorption and degradation in GAC-based treatment systems.

## Abstract 2

14C-labeling is applied in a new approach to confirm degradation of micropollutants in GAC-filter 16 media with potential to optimize future filter design.

## 1 Introduction

Adsorption onto activated carbon is one option for the abatement of organic micropollutants in municipal wastewater. Activated carbon can be applied in powdered (PAC) or granular (GAC) form, depending on the desired treatment configuration.^1,2^ GAC has particle sizes that are 10–100-fold larger than those in PAC and is more susceptible to mass transfer limitations, such as pore-blocking effects.^3,4^ Further, GAC filters are typically operated over long periods, allowing the colonization of microorganisms,^5^ which inevitably results in biofilm formation on the surfaces of GAC particles.

The presence of biofilms on GAC limits the transport of the substrate to the carbon surface,^6^ which could impact the choice of relevant empty bed contact times (EBCTs) for the filter. At the same time, the presence of biofilms allows for the potential long-term degradation of dissolved organic matter^7^ and micropollutants,^8^ which could lead to a partial bioregeneration of the activated carbon adsorption capacity.^9,10^

The large number of bed volumes that can be treated by GAC filters without deteriorating removal of certain organic micropollutants has raised the question of whether biological degradation contributes to long-term GAC-filter performance.^8,11,12^ However, assessing the degradation that occurs in GAC filters is challenging due to the potential adsorption of target micropollutants and their transformation products. The differentiation between biological degradation and adsorption in GAC filters, based on influent–effluent measurements of parent compounds, has been reported to be limited.^13^

Thus far, the contributions of degradation by GAC biofilms to the overall micropollutant removal process have mainly been estimated by comparing removal efficiencies for biologically activated GAC filters with those of sterilized GAC filters.^14−16^ Although this approach has illustrated the enhanced removal of several substances by biologically active GAC filters, this method does not necessarily allow for the strict separation between adsorption and biodegradation.^14^

The use of ^14^C-labeled micropollutants enables adsorption to be separated from degradation because only the latter contributes to the formation of ^14^CO_2_. The simultaneous tracking of ^14^C decay in the water and gas phases (using CO_2_-traps) has been used to study the biological degradation of organic micropollutants in wastewater^17,18^ and drinking water.^19^ The approach has further been used to demonstrate biodegradation and bioregeneration in GAC-filter columns^20,21^ and the potential for increased adsorption of nonbiodegradable compounds through the degradation of biodegradable compounds in two-component systems.^22^ Based on these findings, it appears possible to use ^14^C-labeling to study the degradation of organic micropollutants in wastewater in contact with GAC-filter media.

Therefore, the objective of this study was to use ^14^C-labeled micropollutants to investigate the adsorption and degradation of selected micropollutants in contact with a mature GAC-filter media and to compare the biodegradation potential of GAC-bound biofilms with those from other biofilm and suspended growth processes in a comprehensive set of batch experiments.

## 2 Materials and Methods

### 2.1 Media for Batch Experiments

**Figure 1:**
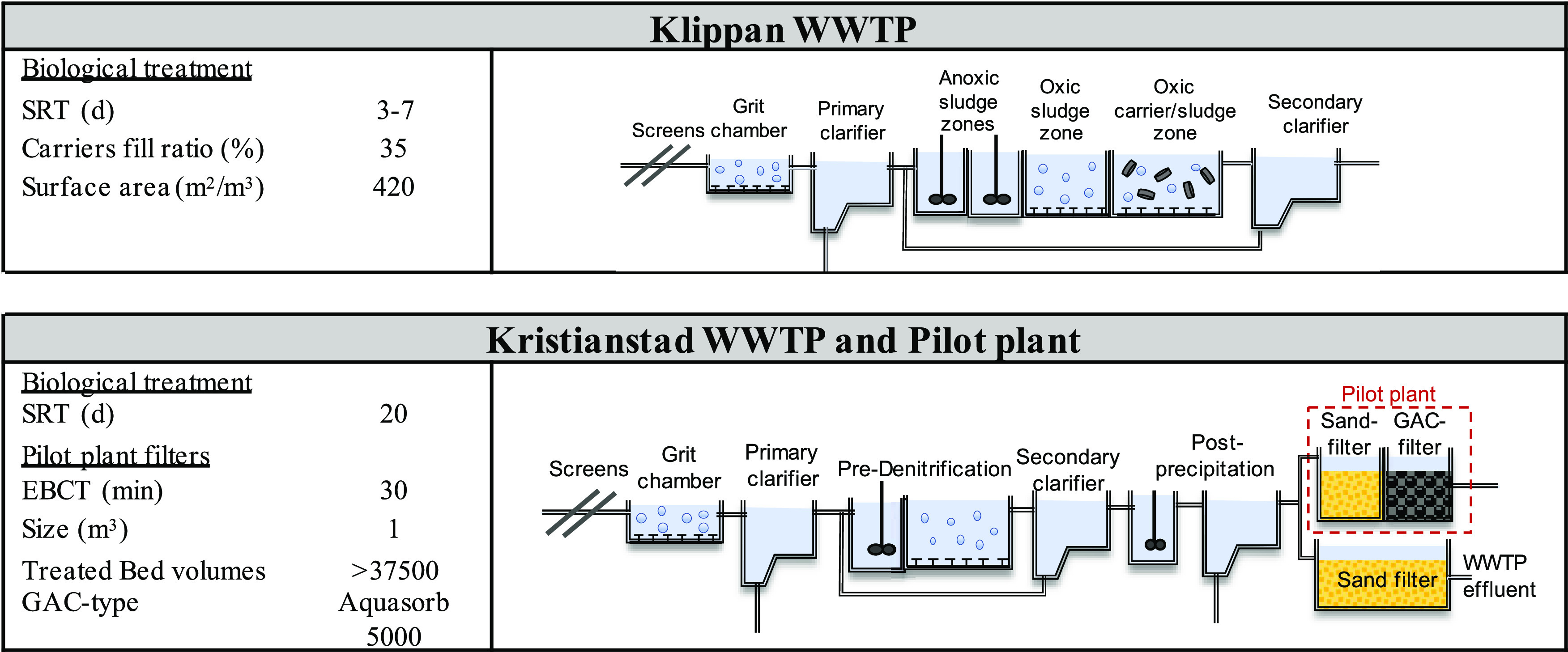
Overview of wastewater treatment plants, including the pilot plant at Kristianstad WWTP.

Laboratory-based experiments were performed on three types of biofilm media: a GAC-filter media, a sand filter media, and carriers from a moving bed biofilm reactor (MBBR). The degradation capacities of each medium were further compared with their corresponding behaviors in an activated sludge process. The biomass media originated from two Swedish wastewater treatment plants (WWTPs), Klippan WWTP and Kristianstad WWTP, and a pilot plant that was operated at Kristianstad WWTP. Overviews of the two treatment plants are provided in Figure 1 and detailed in the Supporting Information (Section S1).

The pilot plant at Kristianstad WWTP treats postprecipitated wastewater and consists of a sand filter and a subsequent GAC filter. The filters are identical in size, 1 m^3^ each, and are operated in downflow mode, with EBCTs of 30 min each. The GAC-filter media was Aquasorb 5000, 8′30 mesh (2.36–0.60 mm, Jacobi), with a specific surface area, according to the Brunauer–Emmett–Teller (BET) theory, of 1200 m^2^/g. At the time of initial experiments with the GAC-filter media, the filter had treated a total of 37 500 bed volumes.

Sand and GAC media were retrieved from the tops of respective filters at the pilot plant, operated at Kristianstad WWTP. To separate the media from the suspended biomass that originated from previous treatment steps, the sand and GAC media were repeatedly washed with effluent pilot wastewater, until the water phase was visibly clear of all particles. The sand and GAC media were stored (<48 h) in effluent wastewater at 8 °C until the start of the experiment. Activated sludge from Kristianstad WWTP and carriers from Klippan WWTP were retrieved the day before the start of the experiment and were aerated overnight.

### 2.2 Micropollutant Selection

**Figure 2:**
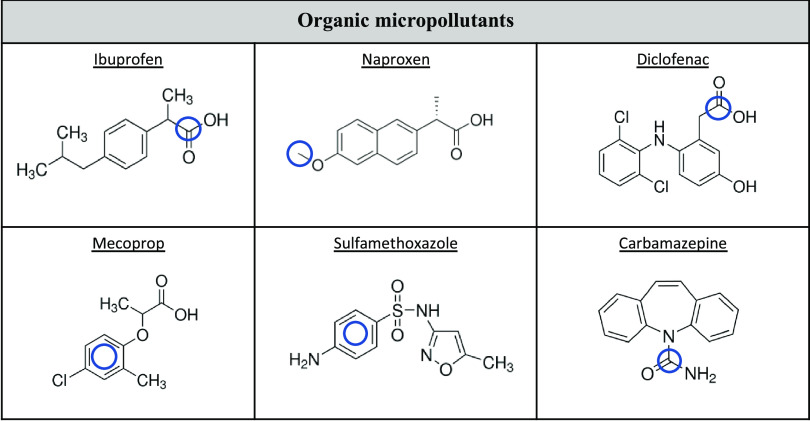
Investigated micropollutants with the ^14^C-labeling indicated by blue circles.

Six ^14^C-labeled micropollutants with varying physicochemical properties were selected (Figure 2): Mecoprop [ring-u-^14^C] and sulfamethoxazole [phenyl ring-u-^14^C], from Izotop (Hungary); and ibuprofen [RS-carboxyl-^14^C], naproxen [*O*-methyl-^14^C], diclofenac [carboxyl-^14^C], and carbamazepine [carbonyl-^14^C], from Hartmann Analytics (Germany). All ^14^C positioning was chosen based on commercial availability. The radiochemical and chemical purities were >98%.

### 2.3 Adsorption and Degradation Experiments

The adsorption and degradation experiments (Table 1) were performed in 500 mL glass bottles containing 150 mL biologically treated and filtered (0.45 μm cellulose nitrate, Whatman) wastewater, the various tested media (sand, GAC, carriers, or activated sludge), and ^14^C-labeled micropollutants. Each micropollutant was studied separately in a biologically active reactor with a corresponding heat-treated (85 °C, 60 min) control and a background control (containing only filtered wastewater). The biologically treated wastewater used for the experiments was retrieved from a separate plant (Lundåkra WWTP), with stable effluent nitrogen and chemical oxygen demand (COD) concentrations, as described elsewhere,^23^ to allow for a better comparison between media. The filtered wastewater added to the reactors was adjusted to pH 7.0, using 10 mM NaH_2_PO_4_ buffer (adjusted with 1 M NaOH), and saturated with oxygen (8–9 mg/L) to prevent anoxic conditions. ^14^C-labeled micropollutants were added at 1 μCi/L, corresponding to concentrations of approximately 6 μg/L mecoprop, 13 μg/L sulfamethoxazole, 4 μg/L ibuprofen, 5 μg/L naproxen, 5 μg/L diclofenac, and 11 μg/L carbamazepine.

**Table 1:**
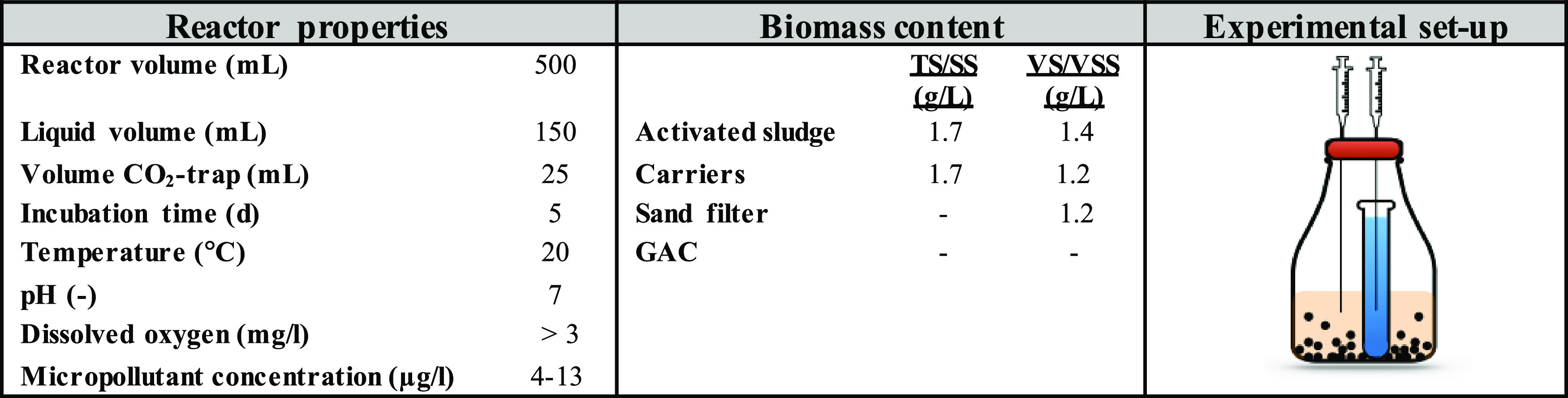
Overview of Treatment Conditions and the Experimental Setup

After the addition of the biomass media, the reactors were sealed immediately with a rubber septum and incubated at 20 °C, with agitation at 150 rpm, for 5 days. To capture formed ^14^CO_2_, a glass tube that contained 25 mL of NaOH (0.1 M) was fixed inside the glass bottle, using a 3D-printed holder. Dissolved oxygen (DO) contents and pH were determined in ^14^C-free controls to ensure aerobic conditions (DO > 3 mg O_2_/L) and neutral pH (7.0–7.4). A more detailed summary of the experimental conditions is provided in Table S1.

Samples from the water phase (1 mL) and the NaOH trap (0.5 mL) were retrieved at regular intervals, through a rubber septum and using hypodermic needles, and were transferred to Eppendorf tubes. To separate the remaining biomass media, water phase samples were immediately centrifugated at 13 000 rpm for 5 min, followed by the transfer of supernatants (0.8 mL) to new Eppendorf tubes.

### 2.4 Degradation of Previously Adsorbed Micropollutants

To further study the interaction between adsorption and degradation, a variation of the previous experiment was designed. During this experiment, the wastewater that was used in the reactor was first purged with N_2_ gas to deplete oxygen, <0.1 mg O_2_/L. Using the same setup as described for the previous experiment, GAC was then allowed to adsorb ^14^C-labeled micropollutants for 24 h, under anaerobic/anoxic conditions, to minimize degradation. After the 24 h anoxic/anaerobic period, samples were taken from the water phase and the CO_2_ trap to estimate the extent of GAC adsorption. The preloaded GAC from the anoxic/anaerobic incubation was then separated from the wastewater by carefully decanting the water. Aerated wastewater, >8 mg O_2_/L, and new CO_2_ traps were then introduced to the reactors, before sealing and incubation as described for the previous experiment (5 days, 20 °C at 150 rpm).

### 2.5 Analysis

The amount of ^14^C originating β-decay was quantified by liquid scintillation counting (Tri-Carb 4910 TR, PerkinElmer). Portions of the samples (0.2 mL of the NaOH trap samples and 0.4 mL of the liquid samples) were mixed with a scintillation cocktail (Hionic-Flour, PerkinElmer), at a total volume of 4 mL, after which the mean numbers of counts per minute (over 5 min) were recorded. The background radiation values measured for wastewater and NaOH (mean of five samples) were subtracted from each sample value.

The main parameters of the biologically treated wastewater samples were measured after filtration (0.45 μm cellulose nitrate, Whatman). The spectrophotometric determination of concentrations was performed on a Hach-Lange DR 2800 using Hach-Lange cuvettes: chemical oxygen demand (COD, LCK 1414), total organic carbon (TOC, LCK 385), NH_4_-N (LCK 303), and NO_3_-N (LCK 339). Ultraviolet (UV) absorbance, at 254 nm (UVA254), was recorded (5 cm quartz cuvettes) using a UV spectrophotometer (Dr6000, Hach).

### 2.6 Biomass Estimation

A target biomass reactor concentration of 1.2 g of volatile solids (VS)/L was selected for the sand filter media and carriers, allowing oxic conditions, >3 mg O_2_/L, to prevail during the 5 day incubation. For the sand filter media, the VS concentration was determined by the dry weight difference before and after burning (550 °C, 60 min). For the carriers, the VS concentration was estimated based on the VS to total solids (TS) ratio and the amount of TS on the carriers. TS concentrations were first determined as the differences in carrier dry weights (105 °C) before and after the careful biofilm removal. The VS-to-TS ratio was then determined by burning the abraded and dried biofilm. For the activated sludge, the biomass concentration was determined as suspended solids (SS) and volatile suspended solids (VSS).

In the GAC experiments, the relative amounts of biomass could not be determined; therefore, a total of four GAC concentrations (0.8, 3.5, 12, and 51 gTS/L) were tested. The lowest dose (0.8 g/L), thus, corresponded to a total weight (biomass + GAC) below the concentrations (1.2 gVS/L or 1.4 gVSS/L) that were used for the other biomass media. Specific biomass/media concentrations were estimated based on 3–5 replicates and are shown in Table S1.

## 3 Results and Discussion

The rates and extent of biological degradation and adsorption in GAC-filter media were investigated and compared with other biofilm systems using ^14^C-labeled organic micropollutants.

### 3.1 Incubation Experiments

#### 3.1.1 Sand Filter Media and Carrier-Attached Biofilm

In biological incubations containing biofilms, the removal of nonvolatile micropollutants can occur through adsorption and degradation via both biotic and abiotic pathways. However, background control experiments using 0.45 μm filtered wastewater (Figure S1) demonstrated negligible changes in ^14^C activities in the liquid and gas phases, which suggested that abiotic transformations occurring in the water phase, as well as biological transformation by microorganisms not retained by the 0.45 μm filtration, have minor influences on the ^14^C mass balance. The lack of ^14^CO_2_ formation in heat-treated controls (Figure S2) also suggested a negligible abiotic transformation induced by reactive surface functionality present on solids. The minor concentration changes that occurred in the liquid phases of heat-treated controls (Figure S2) further indicated negligible adsorption, as expected from low-solid, water partitioning coefficients for suspended sludge of <80 L/kgSS^24,25^ and the applied biomass concentrations, <2 g biomass/L (Table S1).

**Figure 3:**
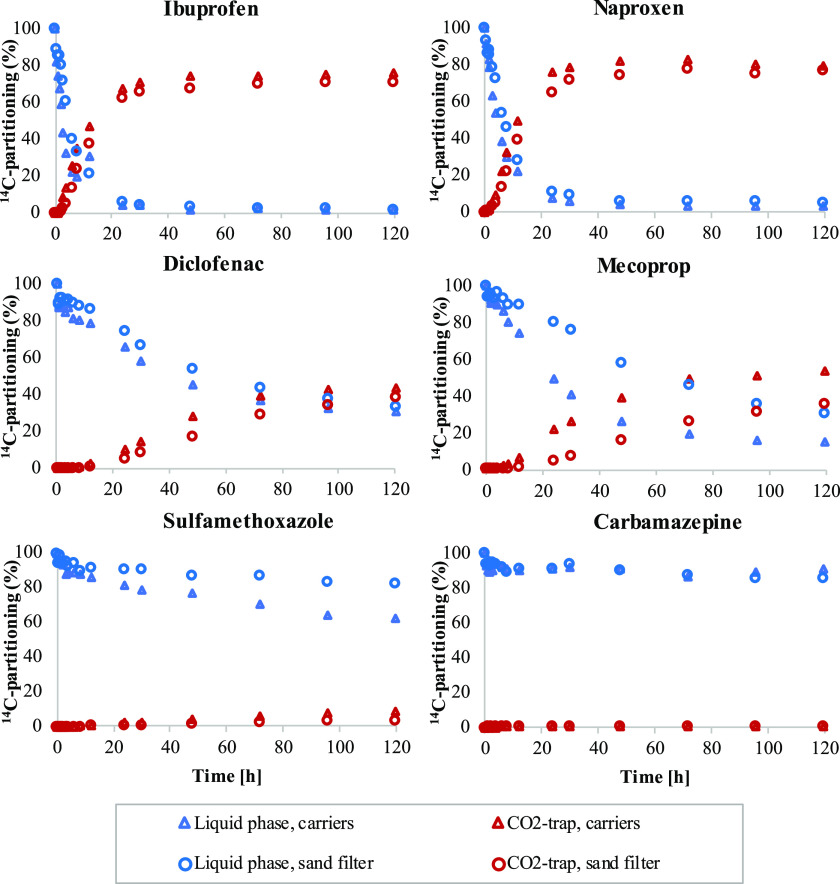
Partitioning of ^14^C activities between liquid phases (blue) and CO_2_ traps (red) for six ^14^C-labeled micropollutants in contact with MBBR carriers (triangles) and the sand filter (circles).

In the incubations with biologically active carrier and sand filter media, the decreasing ^14^C activities in the liquid phase were accompanied by the formation of ^14^CO_2_ (Figure 3). The phase-transfer rates were comparable for both the carriers and the sand filter media. The highest rates were observed for ibuprofen and naproxen, followed by mecoprop, diclofenac, sulfamethoxazole, and carbamazepine.

Ibuprofen and naproxen showed comparable removal rates among biomass that was attached to carriers and sand filter media and in suspended biomass (Figures 3 and S3). The ^14^CO_2_ formation rates were also similar between these two compounds, although ibuprofen tends to be more readily biotransformed,^25,26^ which may be due to the position of the ^14^C-labeling and the transformation pathways of the compounds. Naproxen has been observed to undergo O-demethylation, which targets the ^14^C-labeled position.^27^ In contrast, the parent degradation of ibuprofen occurs through several transformation reactions,^28^ which do not primarily target the ^14^C-labeled carboxylic group.

Diclofenac and mecoprop showed comparable removal rates for carrier-attached biomass and sand filter media, with slightly lower rates for activated sludge (Figures 3 and S3). The parent transformation of diclofenac has been reported to proceed at higher rates with the carrier-attached biofilm compared with suspended sludge.^29,30^ Several primary and secondary transformation products have also been identified, and one of the four primary transformation reactions targeted the ^14^C-labeled carboxylic group.^31^ The potential degradation pathways of mecoprop include the formation of 4-chloro-2-methylphenol and 4-chloro-2-methylphenol sulfate, and the partial transformation of the ^14^C-labeled phenolic ring structure has been demonstrated.^32^

The degradation rates of carbamazepine and sulfamethoxazole were low or negligible. For carbamazepine, the lack of ^14^CO_2_ formation that was observed in all experiments agrees with previous studies, in which no removal has been observed.^33,34^ For sulfamethoxazole, in contrast, the degradation of the parent compound has frequently been observed.^35,36^ However, the transformation pathways seldom include cleavage of the ^14^C-labeled aniline structure, as confirmed in studies examining ^14^C-labeled sulfamethoxazole and activated sludge.^36,37^ However, the degradation of the aniline structure has been reported during long-term incubations (>1 week) with bacterial strains that are isolated from suspended growth systems.^38^

The observed formation of ^14^CO_2_ did not fully match the observed decreases of ^14^C activity in the liquid phase (Figures 3 and S3). Similar observations have been reported for incubations with ^14^C-labeled micropollutants and soil,^39^ biofilm carriers,^32^ and sand filter media.^19^ Control experiments with carriers and acidification (pH 3) at the end of the incubation period resulted in no or negligible ^14^CO_2_ formation (<2% for all compounds) from precipitated ^14^C-carbonate. The missing ^14^C in this study may be due to the adsorption of transformation products or the incorporation of ^14^C into the biomass,^18^ but was not confirmed via the analysis of solid-phase ^14^C activities.

#### 3.1.2 GAC-Filter Media

**Figure 4:**
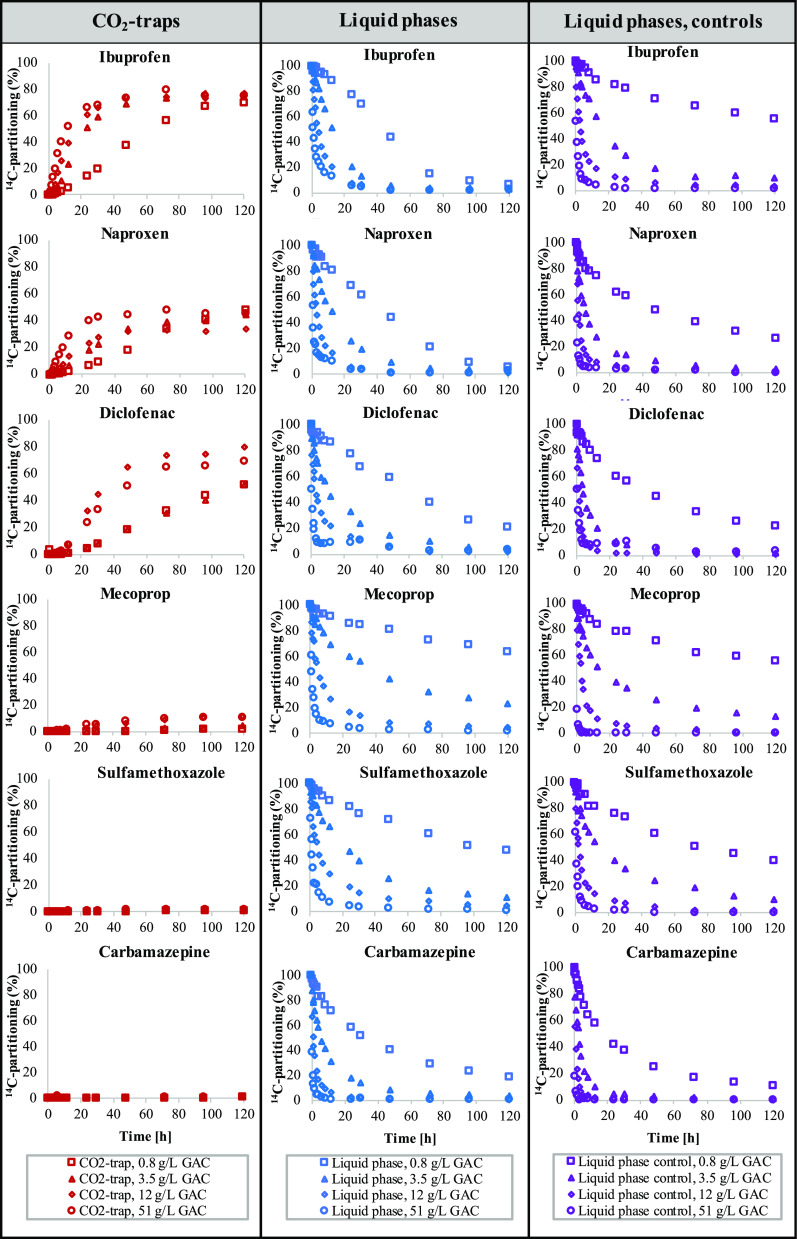
Partitioning of ^14^C activities between CO_2_ traps (red, left panel) and liquid phases (blue, middle panel) for six ^14^C-labeled micropollutants in contact with four different concentrations of GAC-filter media (0.8, 3.5, 12, and 51 gTS/L). The liquid phases of the corresponding heat-treated GAC controls are also shown (purple, right panel).

The results of the degradation and adsorption experiments performed using four concentrations of GAC (0.8, 3.5, 12, and 51 gTS/L) are summarized in Figure 4, with ^14^C activities divided between the CO_2_ trap (left panels), the liquid phase (middle panels), and the liquid phase of the heat-treated control (right panels). The absence of ^14^CO_2_ formation in heat-treated GAC controls (Figure S4) indicated that the observed decreases in ^14^C activities were due to the adsorption of micropollutants. In experiments with the lowest doses of heat-treated GAC, the activity in the liquid phase decreased continuously throughout the experiment, indicating that the observed adsorption profiles were affected by both the adsorption capacity and the adsorption kinetics.

The highest affinity for adsorption onto GAC was observed for carbamazepine, followed by diclofenac and naproxen, and lower affinity for ibuprofen, sulfamethoxazole, and mecoprop. These adsorption patterns are similar to previous results on tertiary PAC treatment, which demonstrated a high level of adsorption for carbamazepine and lower levels of adsorption for sulfamethoxazole and mecoprop.^1,40^

In the experiments containing biologically active GAC, decreasing ^14^C activities in the liquid phase were accompanied by the formation of ^14^CO_2_ for four of the six tested micropollutants (left panel, Figure 4). Degradation, therefore, appears to contribute to the removal of ibuprofen, naproxen, diclofenac, and, to some extent, mecoprop. The formation of ^14^CO_2_ was observed for ibuprofen, naproxen, and diclofenac in all biologically active GAC experiments, and the formation rates generally increased with higher GAC concentrations, as expected due to the increasing amount of GAC-attached biofilm. These three compounds were also transformed in the experiments performed using biofilms attached to carriers and sand filter media (Figure 3). By comparing removal efficiencies between biologically activate and sterilized GAC filters, Rattier et al.^14^ indicated an additional biological removal of 10–20% for the same compounds. The degradation of these three compounds has further been demonstrated by individual γ proteobacteria that has been isolated from a GAC biofilm.^41^ For mecoprop, ^14^CO_2_ formation was only detectable at the highest GAC concentrations but remained below 10%.

The transformation of sulfamethoxazole and carbamazepine could not be detected via the formation of ^14^CO_2_. The declining ^14^C activities in the liquid phase that were observed for sulfamethoxazole and carbamazepine, therefore, appear to be associated with adsorption only. Although carbamazepine is considered to be readily adsorbed^42^ and practically nondegradable,^43^ the degradation of sulfamethoxazole has been indirectly demonstrated previously using GAC-filter media.^16^ As discussed previously, the absence of ^14^CO_2_ formation may be associated with the location of the ^14^C-labeled moiety.

The GAC-bound biofilm was capable of partially degrading the same substances as those degraded by other biofilm processes. A direct comparison of degradation rates between biofilm systems could not be performed due to the unknown biomass concentrations in the GAC experiments. However, the lowest GAC concentration (0.8 g/L) had a lower total weight (GAC + biomass) than the biomass carriers and sand filter media (1.2 gVS/L) but was still able to degrade diclofenac to a greater extent. These results indicated that the GAC biofilm was more efficient for diclofenac degradation than the MBBR carrier and sand filter biofilms. Based on these observations, it appears interesting to further explore the degradation capability of organic micropollutants by the GAC-bound biofilm and to compare degradation rates with other biofilms in a quantitative manner (e.g., using ATP^44,45^ or phospholipid analysis^46,47^).

When liquid phase ^14^C activities in biologically active reactors were compared with heat-treated GAC controls (middle panel and right panel, Figure 4), more rapid adsorption of liquid-phase ^14^C activities was generally observed in heat-treated controls, as illustrated by the faster removal of all substances at high GAC doses (particularly for nondegradable carbamazepine). The higher affinity for adsorption was likely caused by the heat-induced desorption of organic matter from the GAC during the sterilization (85 °C, 60 min) phase, liberating additional adsorption sites and/or decreasing mass transfer resistance. As a result, comparisons between the liquid-phase concentrations could not be used to estimate the overall contribution of biological degradation. However, experiments using the lowest doses of biologically active GAC (0.8 g/L) demonstrated larger decreases of ^14^C activities in the liquid phase for ibuprofen and naproxen compared with the corresponding controls (Figure S4), demonstrating that biological activity increased the overall removal of these pollutants. For higher GAC doses, similar comparisons could not be performed, as the decrease in liquid-phase concentrations always approached 100%.

For biologically active GAC reactors, the decrease of the ^14^C activity in the liquid phase was always more rapid than the corresponding ^14^CO_2_ formation, indicating that adsorption was faster than degradation and that previously adsorbed micropollutants could be degraded at a later time point. The latter phenomenon was particularly notable for diclofenac and could be observed by comparing ^14^C partitioning after 8 h (liquid: 22%; gas: 8%; remaining: 70%) with the corresponding values at the end of the experiment (liquid: 1%; gas: 80%; remaining: 19%). However, some delay in the mass transfer of ^14^CO_2_ from the liquid phase to the CO_2_ trap may also occur.

### 3.2 Degradation of Previously Adsorbed Micropollutants

To further investigate possible interactions between adsorption, desorption, and degradation, an experiment was designed that included a 24 h anaerobic adsorption phase and a subsequent aerobic desorption and degradation phase. Analysis after the anaerobic adsorption phase indicated that the majority of the ^14^C activities were adsorbed to the GAC, with less than 6% detected in CO_2_ traps (Table S2). The restricted degradation of the targeted micropollutants during anaerobic conditions is further supported by previous studies examining the parent compounds, except for sulfamethoxazole.^48−50^ After the anaerobic adsorption phase, the GAC, including adsorbed micropollutants, was separated from the liquid phase and transferred to a new aerobic reactor containing ^14^C-free wastewater.

**Figure 5:**
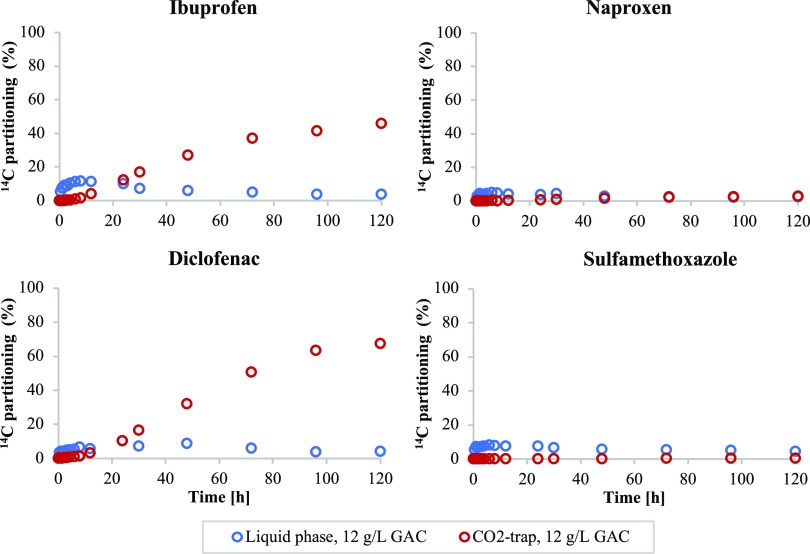
Partitioning of ^14^C activities between the liquid phases (blue) and CO_2_ traps (red) for four previously adsorbed (24 h, anaerobic conditions, 12 g/L GAC) ^14^C-labeled micropollutants.

Figure 5 displays the extent of ^14^CO_2_ formation for the previously adsorbed micropollutants. The results showed that the previously adsorbed ibuprofen and diclofenac could be degraded to 46 and 68%, respectively, illustrating that the mechanisms of removal can occur through the initial adsorption of the compounds followed by their subsequent degradation. Initial desorption could be observed by the increased ^14^C activity in the liquid phase. Whether this initial desorption is a prerequisite for subsequent degradation, however, could not be determined. Nonetheless, the results showed that initial adsorption, followed by degradation, is a possible mechanism for the removal of diclofenac and ibuprofen in GAC filters.

No degradation of previously adsorbed naproxen could, however, be observed after the anaerobic adsorption phase. The lack of CO_2_ formation observed in this GAC experiment was probably not caused by anaerobic inactivation of the biofilm, as the aerobic degradation of naproxen with carriers proceeded at the same rate regardless of the anaerobic pre-exposure (Section S9 and Figure S6). Limited desorption or availability of previously adsorbed naproxen could be an explanation for the lack of degradation, which might be supported by the ceasing ^14^CO_2_ formation as liquid-phase activities approached zero, as shown in Figure 4.

### 3.3 Implications

With respect to the study of biological degradation in GAC processes, the tracking of ^14^C in ^14^C-labeled micropollutants can circumvent some of the inherent limitations of other methods using LC-MS/MS analysis. For instance, estimating the removal of parent compounds through influent–effluent measurements cannot itself explain biological degradation,^13^ and the detection of biological transformation products may be prevented by their potential adsorption onto the activated carbon. While extraction of previously adsorbed micropollutants has been demonstrated,^51,52^ estimating biological contribution based on transformation product extraction still requires extensive knowledge on potential degradation pathways. Furthermore, the inhibition methods used to compare biologically active and sterilized GAC filters^15,53^ may not, selectively or completely,^14^ inhibit biological processes, and observed differences are still difficult to link directly to biological degradation of micropollutants, due to potential changes in activated carbon adsorption capacity induced by degradation of competing natural organic matter.

Despite the advantages of the ^14^C approach, this technique has its own limitations. The applied method can only demonstrate degradation through mineralization (to ^14^CO_2_) of labeled ^14^C-moieties, whereas the partial degradation of ^14^C-labeled moieties, or any partial/complete mineralization of any nonradiolabeled moieties, will pass unnoticed. Nonetheless, the method can enable direct confirmation of biological degradation through the formation of ^14^CO_2_ from ^14^C-labeled moieties as observed for diclofenac, mecoprop, ibuprofen, and naproxen and serve as a complement in future studies on concurrent adsorption and biodegradation in GAC filters.

With the degradation of previously adsorbed diclofenac and ibuprofen, our study has demonstrated the potential interaction between the two processes. This finding strengthens the hypothesis that the biological degradation in GAC filters is not limited by the hydraulic retention time. The potential decoupling of the hydraulic retention time from the micropollutant degradation time could be an important factor in the future design and operation of GAC systems.

## Supporting Information Available

Information on wastewater treatment plant configurations and anaerobic/anoxic control experiments with naproxen; tables show a summary of experimental parameters and additional details for the experiments with initial adsorption and subsequent degradation and anaerobic/anoxic control experiments; figures show ^14^C partitioning in experiments with ^14^C-labeled micropollutants for background control experiments, heat-treatment control experiments, anaerobic/anoxic control experiments, activated sludge experiments, and an additional figure with liquid phase ^14^C activities in GAC experiments with/without heat treatment (PDF)

## Abbreviations

GAC: granular activated carbon
PAC: powdered activated carbon
MBBR: moving bed biofilm reactor
EBCT: empty bed contact time
WWTP: wastewater treatment plant
IFAS: integrated fixed-film activated sludge
SRT: sludge retention time
COD: chemical oxygen demand
BOD: biological oxygen demand
TOC: total organic carbon
BET: Brunauer–Emmett–Teller
DO: dissolved oxygen
UVA254: ultraviolet absorbance at 254 nm
TS: total solids
VS: volatile solids
SS: suspended solids
VSS: volatile suspended solids
LC: liquid chromatography
MS: mass spectrometry
