# Supplementary material for: Tracking 14C-Labeled Organic Micropollutants to Differentiate between Adsorption and Degradation in GAC and Biofilm Processes
Source: Environ Sci Technol. 2021 Jul 27;55(16):11318–27. doi: 10.1021/acs.est.1c02728 (PMC8383275; doi:10.1021/acs.est.1c02728)
Supplement: Supplementary file 1 — es1c02728_si_001.pdf [file es1c02728_si_001.pdf]

Supporting information

Tracking ^{14}C-labeled organic micropollutants to differentiate between adsorption and degradation in GAC and biofilm processes

Alexander Betsholtz[†*], Stina Karlsson[†‡], Ola Svahn[§], Åsa Davidsson[†], Michael Cimbritz[†], Per Falås[†]

[†] Department of Chemical Engineering, Lund University, 221 00 Lund, Sweden
[‡] Sweden Water Research AB, Ideon Science Park, Scheelevägen 15, 223 70 Lund, Sweden
[§]School of Education and Environment, Division of Natural Sciences, Kristianstad University, 291 88 Kristianstad, Sweden

[*]Corresponding author (alexander.betsholtz@chemeng.lth.se)

Table of contents

S1. Wastewater treatment plants **S2**

S2. Summary of experimental parameters **S3**

S3. Background controls **S4**

S4. Heat-treatment controls: carriers, sand-filter and activated sludge **S5**

S6. Activated sludge **S6**

S5. Heat-treatment controls: GAC-filter media **S7**

S7. GAC with/without heat treatment **S8**

S8. Degradation of previously adsorbed micropollutants **S9**

S9. Influence of anaerobic exposure on the degradation of naproxen with MBBR carriers **S10**

Number of pages: 10

Number of figures: 6

Number of tables: 3

S1. Wastewater treatment plants

Klippan WWTP, in Sweden, receives domestic wastewater in the amount of 13,000 connected person equivalents. Incoming water is treated mechanically (screening, grit removal, and sedimentation) and biologically, using pre-denitrification and nitrification. The biological treatment consists of an integrated fixed-film activated sludge (IFAS) process with carriers (BiofilmChip M, 35 % fill-ratio) in the last aerated compartment (Figure 1). The sludge retention time (SRT) ranges from 3-7 days. Phosphorous removal is conducted by simultaneous precipitation during the biological treatment. The treatment results in yearly average effluent values of 17 mg/L COD_{cr}, 2.0 mg/L BOD_7, 10 mg/L N_{tot}, and 0.18 mg/L P_{tot}.

Kristianstad WWTP, also in Sweden, receives municipal wastewater in the amount of 118,000 connected person equivalents. Incoming wastewater is treated mechanically (screening, grit removal, and sedimentation) and biologically, using pre-denitrification and nitrification. The biological treatment is operated as a conventional, activated-sludge process, with a SRT of approximately 20 days (Figure 1). Biologically treated wastewater is then subjected to tertiary treatment, using chemical phosphorous removal and sand-filtration, which results in yearly average effluent values of 30 mg/L COD_{cr}, 2.1 mg/L BOD_7, 7.6 mg/L N_{tot}, and 0.12 mg/L P_{tot}.

S2. Summary of experimental parameters

Table S1 - Summary of experimental parameters.

Experiment	Date	Temp (°C)	Biomass amount (TS/SS /VS/VSS)	GAC/sand dry mass (g/L)	DO (mgO_2/L)	pH (initial/final)	Treated GAC-pilot bed volumes	DOC (mg/L)	COD (mg/L)	NH_4^+-N (mg N/L)	NO_3^- -N (mg N/L)	UVA254 (m^{-1})
**GAC**	2019.03.28	20	-	11.9	> 7.5	7.0–7.4	37 500	9.8	25.3	4.8	0.6	21.4
**GAC**	2019.04.11	20	-	51.4	> 3.9	7.0–7.3	38 200	10.2	26.3	4.6	1.1	23.1
**GAC**	2019.04.26	20	-	3.5	> 8.1	7.0–7.2	38 900	10.9	28.5	1.9	3.9	24.1
**GAC**	2019.05.21	20	-	0.76	> 7.7	7.0–7.1	40 200	10.7	29.7	5.6	0.5	19.3
**Sand filter**	2019.06.13	20	VS: 0.96 g/L	291	> 7.2	7.0–7.3	41 300	9.9	23.9	4.7	0.8	18.5
**GAC (delayed degradation exp.)**	2019.09.26	20	-	13.5	<0.1; >7.5	7.0–7.2	46 300	12.3	30.6	5.22	1.3	23
**MBBR Carriers**	2019.05.15	20	TS: 1.7 g/L<br>VS: 1.2 g/L	-	> 5.6	7.0	-	10.8	29.1	4.8	0.4	17.5
**Activated sludge**	2019.09.19	20	SS: 1.7 g/L<br>VSS: 1.4 g/L	-	> 4.6	7.0-7.1	-	15.8	44.4	4.3	0.2	23.3
**MBBR Carriers**	2021.03.30	20	TS:1.7 g/L<br>VSS: 1.3 g/L		<0.1; >3.2	7.0-7.1	-	10.2	25	1.9	1.7	19.6

TS: total solids**; SS:** suspended solids**; VS:** volatile solids**; VSS:** volatile suspended solids**; GAC:** granular activated carbon**; DO:** dissolved oxygen; **DOC:** dissolved organic carbon**; COD:** chemical oxygen demand; **UVA254;** ultraviolet absorption at 254 nm.

S3. Background controls

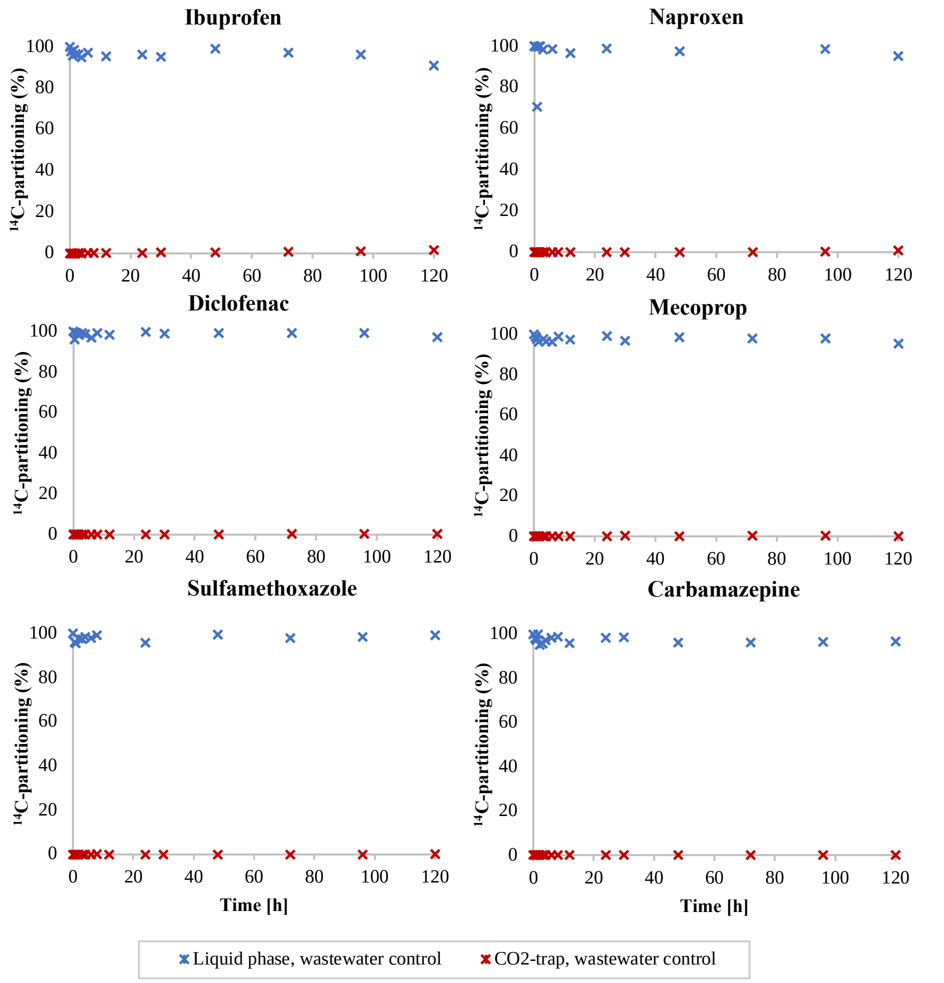

Figure S1 - Partitioning of ^{14}C-activities between the liquid phases (blue) and CO_2 traps (red) for six ^{14}C-labeled micropollutants in contact with filtered (0.45 µm) wastewater.

S4. Heat-treatment controls: carriers, sand-filter and activated sludge

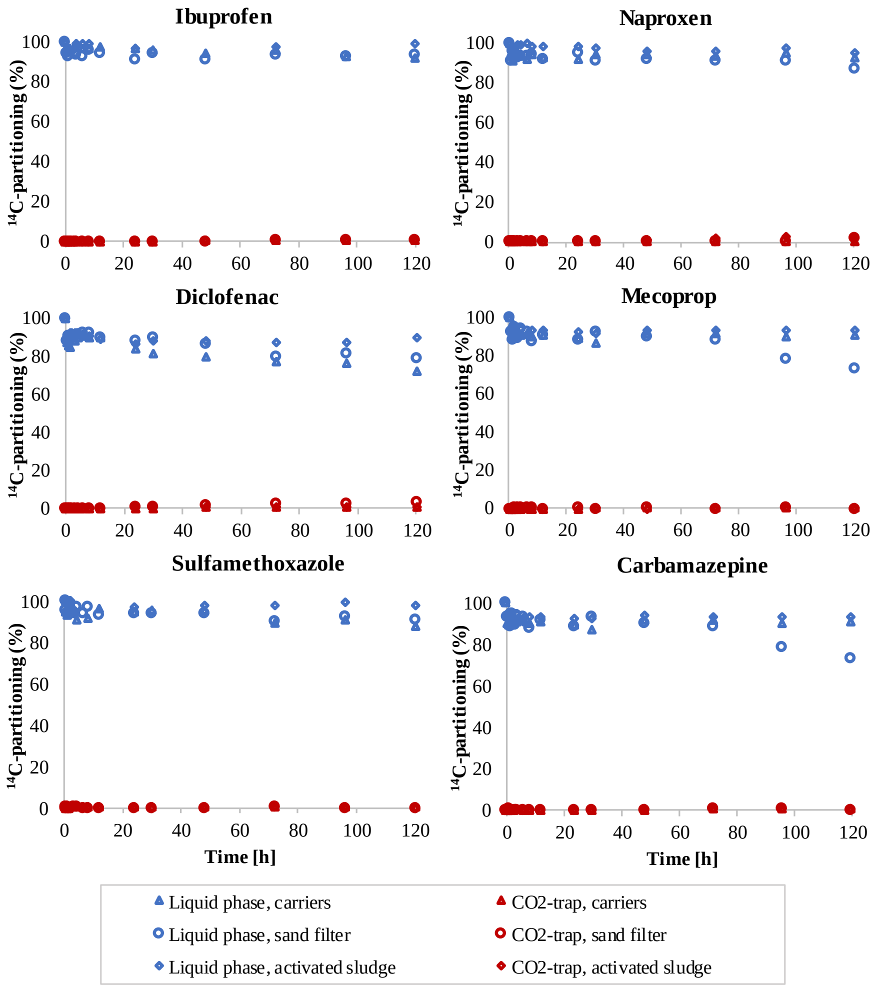

Figure S2 - Partitioning of ^{14}C-activities between the liquid phases (blue) and CO_2 traps (red) for six ^{14}C-labeled micropollutants in contact with the heat-treated controls of the carriers (triangles), sand filter media (rings) and activated sludge (squares).

S5. Activated sludge

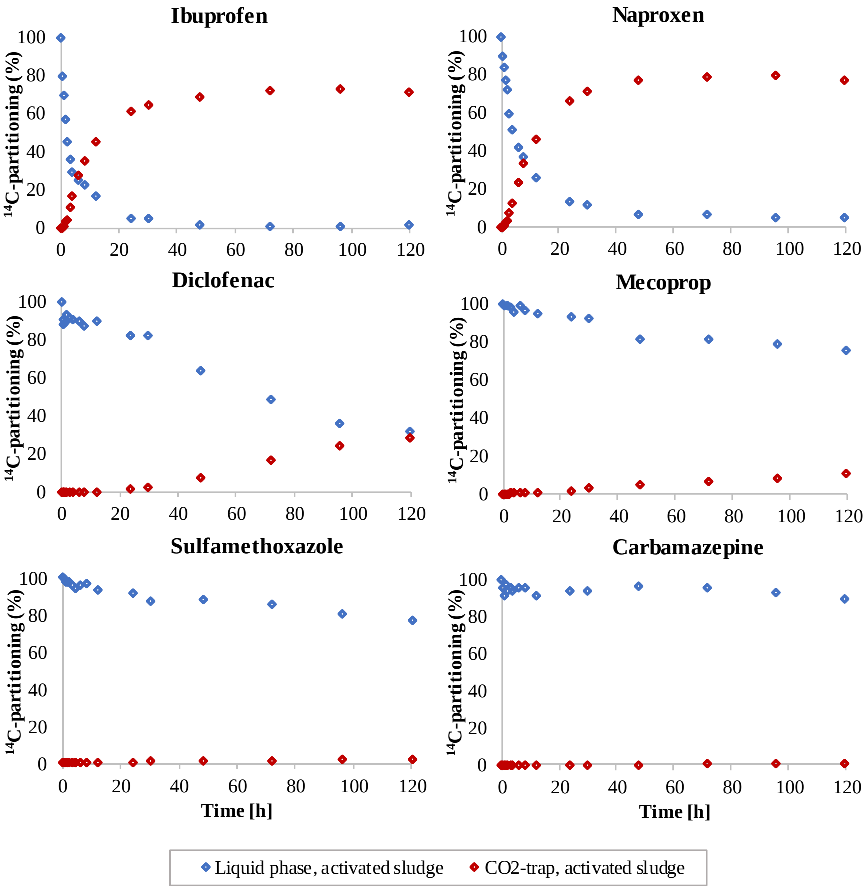

Figure S3 - Partitioning of ^{14}C-activities between the liquid phases (blue) and CO_2 traps (red) for six ^{14}C-labeled micropollutants in contact with activated sludge.

S6. Heat-treatment controls: GAC-filter media

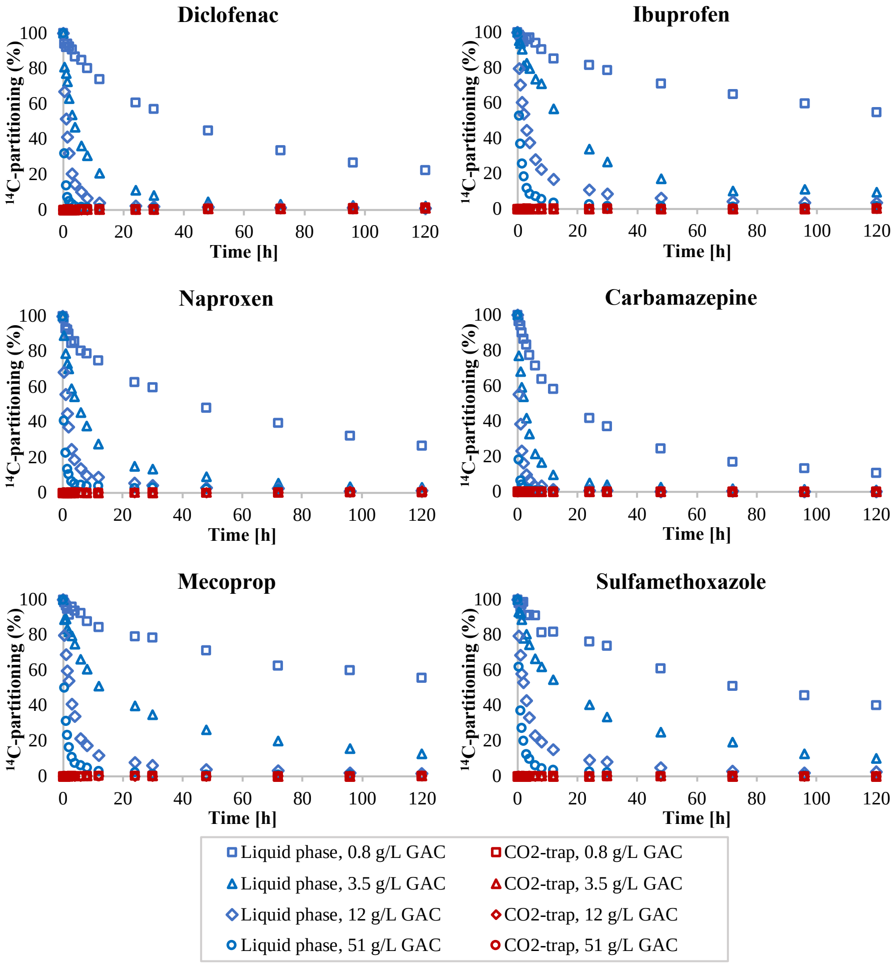

Figure S4 - Partitioning of ^{14}C-activities between the liquid phases (blue) and CO_2 traps (red) for six ^{14}C-labeled micropollutants in contact with the heat-treated controls of four different concentrations of GAC.

S7. GAC with/without heat treatment

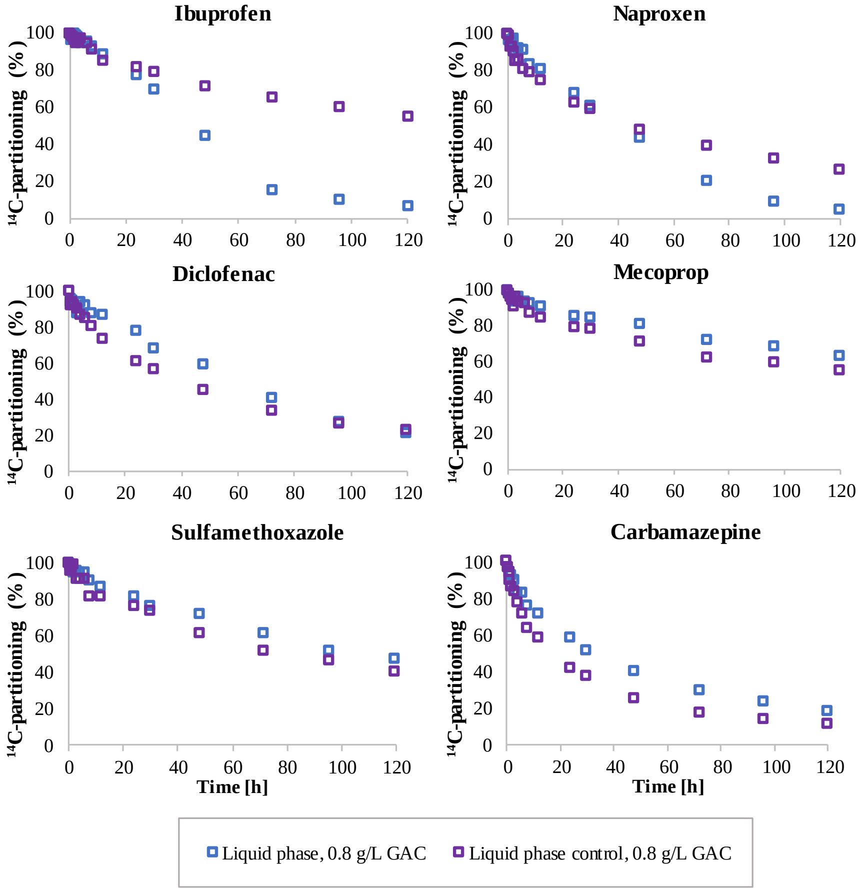

Fig. S5 – Comparison of the partitioning of ^{14}C-activities in the liquid phases for six ^{14}C-labeled micropollutants in contact with biologically activate GAC (blue) and corresponding heat-treated control (purple) using the lowest GAC concentration (0.8 g/L).

S8. Degradation of previously adsorbed micropollutants

Table S2 - Partitioning of ^{14}C between the liquid phases, CO_2-traps, and remaining (adsorbed) fractions after 24 hours of exposure to anaerobic conditions during the delayed degradation experiments.

	**CO_2-trap (%)**	**Liquid phase (%)**	**% Adsorbed**
**Ibuprofen**	1	30	69
**Naproxen**	1	9	90
**Diclofenac**	6	10	84
**Mecoprop**	0	20	80
**Sulfamethoxazole**	0	21	79
**Carbamazepine**	0	2	98

S9. Influence of anaerobic exposure on the degradation of naproxen with MBBR carriers

Experiment description

To investigate whether the 24-hour anaerobic exposure inhibits subsequent microbial aerobic degradation of ^{14}C-labeled naproxen, the following experiment was designed:

Three different reactors containing MBBR carriers (1.3 gVS/L) and ^{14}C-labeled naproxen were exposed to anaerobic or aerobic conditions for 24 hours and subsequently incubated aerobically for 5 days according to the experimental description in section 2.3. The reactors set-up varied as follows:

- In reactor 1, carriers were exposed to anaerobic conditions (DO <0.1mgO_2/L) for 24 hours with ^{14}C-labeled Naproxen added during the exposure. After 24 hours, samples from the liquid phase and CO_2-trap were retrieved to assess the degree of mineralization of the ^{14}C labeled methyl group during anaerobic conditions. The liquid phase was then sparged with air for 2 minutes, resulting in DO>6 mgO_2/L

- In reactor 2, carriers were exposed to anaerobic conditions (DO <0.1mgO_2/L) for 24 hours without ^{14}C-labeled Naproxen being added. After this period, the liquid phase was carefully removed from the reactor and replaced with aerated (DO> 8mgO_2/L) water containing ^{14}C-labeled Naproxen.

- In reactor 3, carriers were exposed to aerobic conditions (DO> 3mg/L) for 24 hours without ^{14}C-labeled Naproxen being added. After this period, the liquid phase was carefully removed from the reactor and replaced with aerated (DO> 8mgO_2/L) water containing ^{14}C-labeled Naproxen.

Results

Table S3 - Partitioning of ^{14}C activities between the liquid phase and CO_2-trap after 24 hours of anaerobic exposure of MBBR carriers with Naproxen *[O-methyl-^{14}C].*

	CO_2-trap (%)	Liquid phase (%)	Total (%)
**Naproxen**	2	97	99

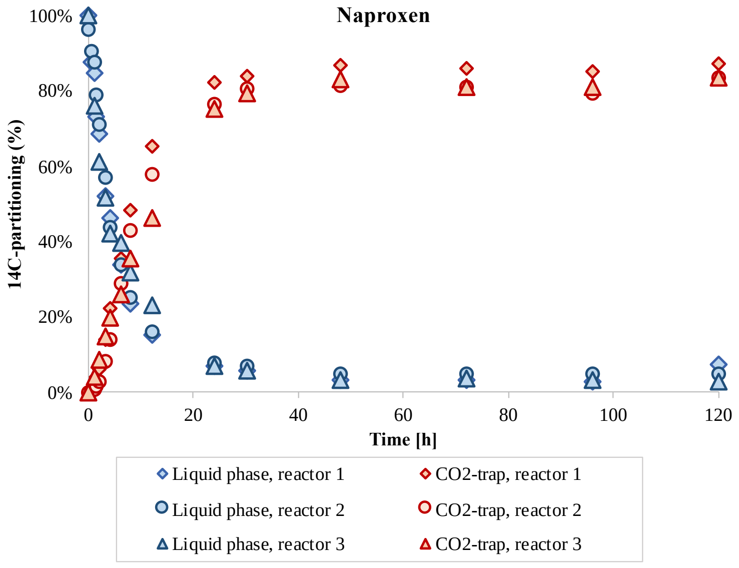

Figure S6 - Partitioning of ^{14}C-activities between the liquid phases (blue) and CO_2 traps (red) subsequent to a 24-hour period with anaerobic conditions (reactor 1 and 2) or aerobic conditions (reactor 3).
